# CPEB4-Dependent Neonate-Born Granule Cells Are Required for Olfactory Discrimination

**DOI:** 10.3389/fnbeh.2019.00005

**Published:** 2019-01-23

**Authors:** Ching-San Tseng, Shen-Ju Chou, Yi-Shuian Huang

**Affiliations:** ^1^Institute of Biomedical Sciences, Academia Sinica, Taipei, Taiwan; ^2^Institute of Cellular and Organismic Biology, Academia Sinica, Taipei, Taiwan

**Keywords:** CPEB4, granule cell, neurogenesis, olfactory discrimination, translational control

## Abstract

The rodent olfactory bulb (OB) contains two distinct populations of postnatally born interneurons, mainly granule cells (GCs), to support local circuits throughout life. During the early postnatal period (i.e., 2 weeks after birth), GCs are mostly produced locally from progenitor cells in the OB with a proportion of them deriving from proliferating cells in the rostral migratory stream (RMS). Afterward, the replenishment of GCs involves differentiated neuroblasts from the subventricular zone (SVZ) in a process known as adult neurogenesis. Although numerous studies have addressed the role of SVZ-born GCs in olfactory behaviors, the function of GCs produced early postnatally in the OB remains elusive. Our previous study demonstrated that the translational regulator, cytoplasmic polyadenylation element-binding protein 4 (CPEB4), is a survival factor exclusively for neonate-born but not SVZ/adult-derived GCs, so CPEB4-knockout (KO) mice provide unique leverage to study early postnatal-born GC-regulated olfactory functions. CPEB4-KO mice with hypoplastic OBs showed normal olfactory sensitivity and short-term memory, but impaired ability to spontaneously discriminate two odors. Such olfactory dysfunction was recapitulated in specific ablation of *Cpeb4* gene in inhibitory interneurons but not in excitatory projection neurons or SVZ-derived interneurons. The continuous supply of GCs from adult neurogenesis eventually restored the OB size but not the discrimination function in 6-month-old KO mice. Hence, in the early postnatal OB, whose function cannot be replaced by adult-born GCs, construct critical circuits for odor discrimination.

## Introduction

Sense of smell allows us to distinguish various odors in the environment and to perceive potential pleasure and danger derived from the odorants. This processed sensory input then triggers behavioral responses (Shepherd, 2004; Feierstein, 2012). Olfaction begins with the stimulation of olfactory sensory neurons (OSNs) in the nasal mucosa. Each OSN expresses only one odorant receptor, and OSNs expressing the same receptor converge onto a few glomeruli in the olfactory bulb (OB), where the synaptic relay is passed onto the projection neurons [i.e., mitral cells (MCs) and tufted cells (TCs)], whose axons transfer the integrated information to several regions in the brain, including the piriform cortex, also called the olfactory cortex (Leinwand and Chalasani, 2011; Mori and Sakano, 2011). Various interneurons fine-tune this relay of olfactory signal, in which the gamma-aminobutyric acid-ergic (GABAergic) granule cells (GCs) in the GC layer (GCL) are the most abundant neurons in the OB. The ratio of GCs to MCs in the OB is ~100:1, much higher than in other parts of the brain (Shepherd and Greer, 1998). GCs form dendrodendritic synapses with MCs and TCs to confine their excitability (Isaacson and Strowbridge, 1998; Schoppa et al., 1998; Takahashi et al., 2016). This lateral inhibition enhances the contrast between activated glomerular units by gating the tuning specificity of projection neurons before sending an integrated signal to the olfactory cortex (Katoh et al., 1993; Yokoi et al., 1995; Xiong and Chen, 2002).

In contrast to other sensations, one unique feature of the olfactory system is its life-long self-renewal of OSNs in the nasal epithelium (Graziadei and Graziadei, 1979) and interneurons in the OB (Hinds, 1968; Ming and Song, 2005; Lledo et al., 2006). OBs receive interneurons, mainly GCs, from two distinct cell sources (Lemasson et al., 2005; Cushman et al., 2012). As embryonic OBs are evaginated from rostral telencephalon, neural precursors derived from the deep ventricular zone (Bailey et al., 1999) continue to proliferate locally and differentiate into GCs after birth, which peaks within the first postnatal week and declines prominently thereafter (Hinds, 1968; Lemasson et al., 2005). During this early postnatal period, neural precursors in the rostral migratory stream (RMS) also migrate to OB and contribute to a percentage of neonate-born GCs (Pencea and Luskin, 2003). Subsequently, neural precursors generated from neural stem cells in the subventricular zone (SVZ) move along the RMS to reach the OB, where they differentiate into GCs and integrate in the existing circuits (Lledo et al., 2006; Grubb et al., 2008). Thus, SVZ-derived GCs, which accumulate approximately after postnatal 2–3 weeks and reach a plateau of ~40% of total GC population in the OB at 3 months (Cushman et al., 2012), continue turnover to maintain and renew the inhibitory circuits throughout life (Imayoshi et al., 2008; Mouret et al., 2009; Lazarini et al., 2014). The two waves of spatiotemporally distinct neurogenesis produce bulbar GCs of high heterogeneity (Kelsch et al., 2007; Batista-Brito et al., 2008; Hardy et al., 2018). Moreover, neonate-born GCs, almost all surviving to adulthood as compared with only ~50% survival of adult-born GCs, are distributed in the superficial part of the GCL closer to MCs (Lemasson et al., 2005), so the local and SVZ neurogenesis likely produce GCs to establish spatiotemporally distinct circuits.

Although the continuous supply of bulbar interneurons is critical to maintaining local inhibitory circuits (Imayoshi et al., 2008), how these newly generated neurons affect olfactory performance and behaviors lacks consensus, likely due to variables in approaches taken to perturb adult neurogenesis in previous studies (Lledo et al., 2006). Because associative olfactory discrimination learning tasks enhance the survival of adult-born GCs (Alonso et al., 2006), SVZ-derived GCs are thought to function in odor discrimination (Lledo et al., 2006). Nevertheless, abolishing the proliferation of neural stem cells in the SVZ by cranial radiation or an antimitotic drug infusion impairs only reward-dependent long-term or spontaneous short-term odor-associated memory, respectively, in adult mice (Breton-Provencher et al., 2009; Lazarini et al., 2009). In contrast to the plentiful studies of SVZ-derived GCs, the physiological function of GCs locally generated in early postnatal OBs remains uncharacterized.

Cytoplasmic polyadenylation element binding protein 4 (CPEB4) is an RNA-binding protein that promotes polyadenylation and translation of target mRNAs (Igea and Méndez, 2010; Novoa et al., 2010). In our previous study, we demonstrated that CPEB4 contributes to olfactory experience-dependent survival of neonate-born GCs. In response to neuronal activity, CPEB4 binds to c-*Fos* mRNA and promotes its translation. In CPEB4-knockout (KO) OBs, reduced c-FOS expression attenuates the transcription of *brain-derived neurotrophic factor* (*Bdnf*), and the insufficient neurotrophic signaling leads to increased apoptotic neonate-born GCs (Tseng et al., 2017). Hence, we used CPEB4-KO mice here to assess the functional importance of neonate-born GCs. Despite their normal olfactory sensitivity and short-term memory, CPEB4-KO mice failed to discriminate a novel odor from a familiar one in the spontaneous odor discrimination test. We further assessed odor discrimination ability in mice with conditional KO (cKO) of CPEB4 in bulbar interneurons, projection neurons or SVZ-derived interneurons, and in 6-month-old CPEB4-KO mice with OB size recovered by the supply of adult-born GCs. Together, the results suggest that GCs born in the early postnatal OB are required for olfactory discrimination.

## Materials and Methods

### Ethical Statement

This study was approved by Institutional Animal Care and Use Committee (IACUC) of Academia Sinica and was compliant with the Taiwan Ministry of Science and Technology guidelines for ethical treatment of animals. All experimental protocols were performed in accordance with the guidelines of IACUC for the ethical treatment of animals and minimized the number of mice used and their suffering. Mice were housed under a 12-h light/dark cycle with lights on at 8:00 in a climate-controlled room with *ad libitum* access to food and water.

### Animals and Genotyping

Generation and characterization of mice carrying the floxed allele (*Cpeb4*^f/f^) or the KO allele of *Cpeb4* in a C57BL/6 genetic background were performed as described (Tsai et al., 2013). *Gad65*-Cre (stock# 019022) and *Gfap*-Cre (stock# 012886) mice were purchased from the Jackson Laboratory. *Nex*-Cre mice were obtained from Klaus-Armin Nave (Max-Planck Institute, Germany; Goebbels et al., 2006). The three CPEB4-cKO mice and their cWT littermates were obtained by mating *Cpeb4*^f/f, +/+^ female mice with *Cpeb4*^f/f, *Nex-cre*/+^ or *Cpeb4*^f/f, *Gad65-cre*/+^ male mice or *Cpeb4*^f/f,+/+^ male mice with *Cpeb4*^f/f, *Gfap-cre*/+^ female mice. CPEB4-WT and -KO mice were littermates from heterozygous matings. The mouse genotype and *Cre* transgene were determined by PCR as described (Tsai et al., 2013; Tseng et al., 2017).

### Immunohistochemistry and Image Acquisition

To limit the circadian effect on bulbar gene expression (Granados-Fuentes et al., 2006), mice were anesthetized and then sacrificed for tissue collection between 14:00 and 16:00 h. Adult male mice (3-month-old) were anesthetized and perfused intracardially with 4% formaldehyde in phosphate buffered saline (PBS). The brain was isolated and further fixed in 4% formaldehyde at 4°C overnight, then dehydrated in 25% sucrose solution. Coronal sections of the OB at 20 μm thick were obtained by using a cryostat (Leica). For antigen retrieval, tissue sections were immersed in 160 ml of 10 mM sodium citrate buffer (pH 6) and heated in a 900W microwave with full power for 2 min and then 20% power for 8 min. OB sections were permeabilized and blocked in PBS containing 0.5% Triton X-100 and 5% bovine serum albumin at room temperature for 1 h, then incubated with primary antibodies against CPBE4 (Tsai et al., 2013) and T-box brain protein 2 (TBR2; Thermo Fisher, Waltham, MA, USA, catalog No. 12-4875-82) at 4°C overnight. After three washes of PBS, sections were incubated with Alexa Fluor-conjugated secondary antibodies and 4’,6-diamidino-2-phenylindole (DAPI) at room temperature for 1 h and then washed with PBS three times before mounting with ProLong Gold Antifade reagent (Invitrogen, Carlsbad, CA, USA). Fluorescence images were acquired by using an Axioimager Z1 upright motorized microscope (Carl Zeiss).

### Olfactory Behavior Assays

The behavior tasks were performed according to published protocols with slight modification (Gheusi et al., 2000; Breton-Provencher et al., 2009). All assays were performed between 14:00 and 18:00 h to limit the circadian effect on mouse behaviors (Granados-Fuentes et al., 2006) and the inter-test interval was at least 2 days to avoid any interference between different behavioral tests. In all cases, the experimenter was blinded to genotype, and tasks involved ~3-month-old female mice unless otherwise specified. Mice were habituated in a polycarbonate cage (275 × 185 × 155 mm) with a glass plate for at least 1 h before the test.

#### Olfactory Sensitivity Assay

Mice were exposed to two filter papers, one saturated with the designated odor mixture (i.e., paprika or cinnamon) and the other with water, placed on the two sides of the glass plate. Three concentrations in a descending order of odor mixtures were used (10^−3^, 10^–4^, and 10^–5^) in separate sessions. Mice were free to explore the scented and non-scented papers for 5 min. The time mice spent sniffing the odor mixture or water control was recorded to calculate the odor detection index as a percentage (investigating time for the odor divided by that for both odor and water × 100). Lack of detection of the odorant stimulus was considered to be when mice spent as much time investigating the odor as the water control, so the index was 50%.

#### Olfactory Short-Term Memory

Mice were exposed to the paprika mixture (10^–3^ w/w) in two 5-min trials with inter-trial interval of 60-, 80-, 100-, or 120-min. The time mice spent investigating the odor mixture was recorded. A significant decrease in odor-investigating time during the second exposure indicated that the animal remembered the odor.

#### Spontaneous Olfactory Discrimination

This assay involved two tasks: in the habituation task, mice were exposed to the first odor (paprika mixture) on one side of the plate and water on the other side for four consecutive 5-min trials with a 15-min inter-trial interval; in the 5th trial, the dishabituation task, mice were exposed to a novel odor (cinnamon mixture) for 5 min. To assay spontaneous discrimination of pure chemicals, (S)-(−)-limonene and 2-heptanolwere diluted in mineral oil to a vapor-phase partial pressure of 1 Pascal before use. The resulting concentrations were 0.436% v/v and 0.838% v/v for (S)-(−)-limonene and 2-heptanol, respectively (Friberg et al., 1998; Devore et al., 2013).

### OB Isolation and Weight Measurement

Mice were anesthetized and then decapitated on ice. After the removal of scalp, the border between parietal and interparietal bones was incised to separate them, followed by cutting the midline of parietal and frontal bones to open the cranium. After removing parietal and frontal bones, both sides of maxillae were crushed and removed carefully by a curved forceps (Fine Science Tools, #11051-10) until revealing the entire OBs. The pair of OBs was isolated by using a fine spatula (Fine Science Tools, #10094-13) to cut the cortical junction and then measured on an analytical balance immediately.

### Data Presentation and Statistical Analysis

All data are expressed as mean ± SEM and were analyzed by GraphPad Prism software. Genotypes and odor exposure trials were compared by Student’s *t*-test and two-way repeated measures ANOVA with Holm-Sidak *post hoc* comparison, respectively. Sample sizes and statistical methods for experiments are in figure legends. *P* < 0.05 was considered statistically significant.

## Results

### CPEB4-KO Mice Exhibited Defective Odor Discrimination

**Figure 1:**
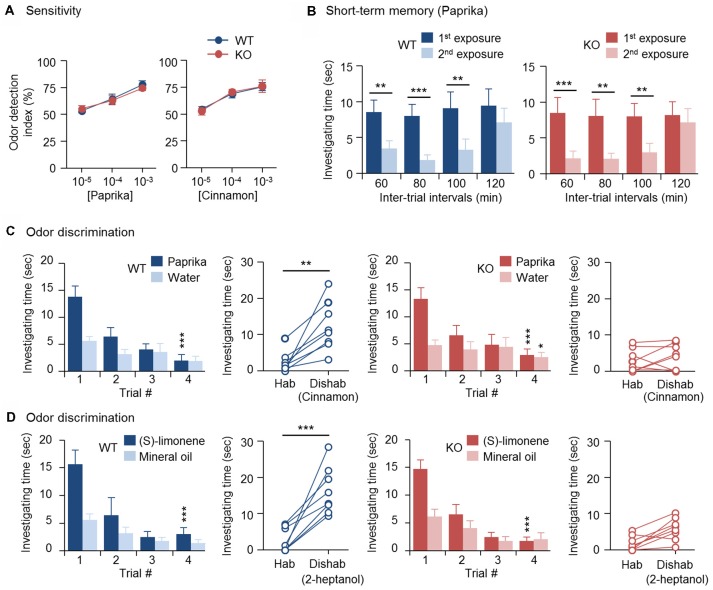
Impaired odor discrimination in cytoplasmic polyadenylation element-binding protein 4-knockout (CPEB4-KO) female mice. CPEB4-WT and -KO female littermates (~3-month-old) were used for assays (*n* = 8 per group). **(A)** Olfactory sensitivity assay. Odor detection index is defined as the percentage of total time (i.e., time to investigate both scented and water papers) mice spent sniffing scented (paprika or cinnamon) paper. **(B)** Olfactory short-term memory assay. The mice were exposed to the paprika odor twice, at the indicated intervals. Each bar represents the mean time spent investigating the odor in a trial. **(C)** Olfactory discrimination assay. Habituation (Hab, bar graphs) test of the mean time mice spent sniffing paprika vs. water for four consecutive trials to become familiar with the odor and gradually reduce investigation interest. Discrimination test: in the 5th dishabituation (Dishab) trial, mice were exposed to a novel cinnamon odor. The time mice spent investigating the two odors was recorded. **(D)** Olfactory discrimination assay. The procedure was identical to **(C)** except using (S)-(−)-limonene and 2-heptanol for habituation and dishabituation test, respectively. Data are mean ± SEM. Student’s *t*-test in the dishabituation task **(C,D)** and two-way ANOVA with Holm-Sidak *post hoc* comparison between the 1st and 2nd exposure in short-term memory **(B)** and between trial #1 and #4 in the habituation task **(C,D)**. *p < 0.05, **p < 0.01, ***p < 0.001.

Previously, we found that OBs of 3-month-old CPEB4-KO mice of both sexes are hypoplastic and contain a reduced number of GCs due to increased apoptosis in only neonate-born GCs (Tseng et al., 2017). Depletion of CPEB4 in GABAergic neurons but not neural stem cells in the SVZ is sufficient to recapitulate such olfactory abnormalities (Tseng et al., 2017). Hence, we used CPEB4-KO mice to determine the olfactory function of neonate-born GCs. CPEB4-KO female mice and their WT littermates were used for the following behavior assays, which were based on the natural instincts of rodents—prefer sniffing scented than non-scented objects, lose interest in investigating a previously experienced odor, and favor a novel to a familiar smell. In the olfactory sensory task, both WT and KO mice spent equally more time on the paper scented with a high concentration (10^–3^ or 10^–4^ w/w) of paprika or cinnamon than the non-scented (water control) paper, so the odor detection index was about 50%; however, both groups failed to recognize scented vs. non-scented paper with a low concentration (10^–5^ w/w; Figure 1A). Thus, the odor detection threshold for paprika and cinnamon was indistinguishable between WT and KO mice.

To evaluate olfactory short-term memory, WT and KO female mice were exposed to paprika twice, separated by an interval of 60, 80, 100 or 120 min (Figure 1B). Within the 100-min interval, both groups of mice spent significantly less time sniffing the paprika-scented paper in the second trial (WT, *F*_(1,21)_ = 41.623, *p* < 0.001; KO, *F*_(1,21)_ = 57.719, *p* < 0.001), because they remembered the given odor in the first trial and lost interest in exploring it. Such an olfactory memory faded at 120 min after the first odor exposure, and both groups of mice regained their interest in sniffing the scented paper in the 2nd trial and spent similar investigating time compared to that in the first trial (WT, *p* = 0.175; KO, *p* = 0.334). Accordingly, CPEB4-WT and -KO mice showed no distinguishable difference in short-term olfactory memory (Figure 1B).

Moreover, we used a spontaneous instead of rewarded paradigm to evaluate olfactory discrimination, so the ability of mice to distinguish two odors did not depend on reward-associated learning. Because both WT and KO mice had normal short-term olfactory memory (Figure 1B), they spent decreasing time investigating paprika-scented paper after four repeated consecutive exposures (WT, *F*_(3,21)_ = 12.264, *p* < 0.001; KO, *F*_(3,21)_ = 16.316, *p* < 0.001); hence, they habituated similarly to the given odor (Figure 1C). CPEB4-WT mice immediately recognized the novel odor and spent more time sniffing it (*p* = 0.002); whereas CPEB4-KO mice were unable to discriminate this novel odor (*p* = 0.51; Figure 1C).

In our previous study, we observed OB hypoplasia in P14 CPEB4-KO male and female mice, so the experiments designed afterward to unravel the molecular defects were conducted in the WT and KO littermates of both sexes (Tseng et al., 2017). Because all anatomical and molecular abnormalities found in CPEB4-KO OBs showed no gender bias, the data obtained from both sexes were analyzed together (Tseng et al., 2017). Nevertheless, it is well recognized that female mammals generally have a better sense of smell than males (Baum and Keverne, 2002; Doty and Cameron, 2009). A recent report indicated that circulating gonadal hormones play a positive and negative role in female and male mice, respectively, by modulating the output activity of OSNs in response to odorant stimulation (Kass et al., 2017). Therefore, female mammals tend to exhibit enhanced sensitivity to odors, in part, due to their estrogen effect (Sorwell et al., 2008; Doty and Cameron, 2009). By contrast, olfactory discrimination appears less relevant to sexual dimorphism. Although male mice outperformed females in the reversal learning of simple odor discrimination task, female and male mice performed comparably in the initial odor discrimination trial (Mihalick et al., 2000). To confirm this, we also performed habituation and dishabituation tests on male mice (Supplementary Figure S1). Consistent with their female littermates, both WT and KO male mice spent decreasing time sniffing paprika-scented paper after four repeated consecutive exposures (WT, *F*_(3,15)_ = 7.284, *p* = 0.003; KO, *F*_(3,15)_ = 11.494, *p* < 0.001); however, in the 5th dishabituation trial, CPEB4-WT male mice recognized the novel cinnamon odor and spent more time sniffing it (*p* = 0.001); whereas CPEB4-KO male mice were unable to discriminate this novel odor (*p* = 0.26; Supplementary Figure S1). Similar to anatomical and molecular defects (Tseng et al., 2017), CPEB4 deficiency caused behavioral abnormality regardless of sex. Therefore, we used female mice for the rest of behavioral assays.

To further verify that the discrimination inability in CPEB4-KO mice does not restrict to a specific pair of odors, CPEB4-WT and -KO female littermates were assayed to distinguish pure chemicals, (S)-(−)-limonene and 2-heptanol. Both groups of mice exhibited similar habituation pattern to (S)-(−)-limonene (WT, *F*_(3,21)_ = 18.223, *p* < 0.001; KO, *F*_(3,21)_ = 19.23, *p* < 0.001; Figure 1D). In the dishabituation trial, WT mice showed more interest to the novel 2-heptanol (*p* < 0.001) but CPEB4-KO mice could not discriminate and spend less time to sniff the novel odor (*p* = 0.25). Thus, CPEB4 deficiency had no influence on olfactory sensitivity and short-term memory but impaired olfactory discrimination.

### Loss of CPEB4 in Early Postnatal Interneurons but Not Projection Neurons Impairs Olfactory Discrimination

**Figure 2:**
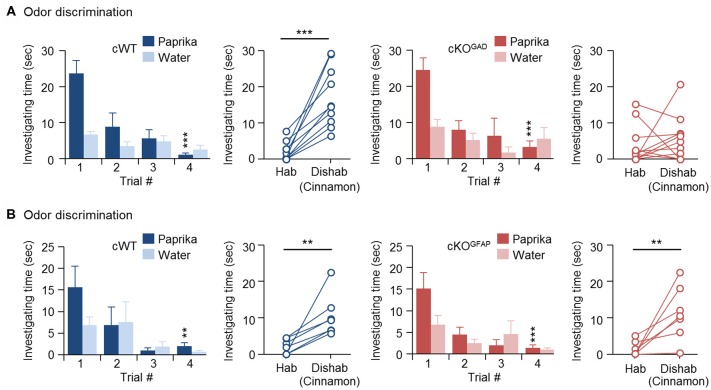
Ablation of *Cpeb4* in gamma-aminobutyric acid-ergic (GABAergic) interneurons but not neural stem cells impairs odor discrimination. ~3-month-old female littermates were used for spontaneous discrimination between paprika and cinnamon odors. **(A)** conditional WT (cWT; *Cpeb4*^f/f,+/+^, *n* = 10) and cKO^GAD^ (*Cpeb4*^f/f,Gad65-cre/+^, *n* = 11) mice. **(B)** cWT (*Cpeb4*^f/f,+/+^, *n* = 7) and cKO^GFAP^ (*Cpeb4*^f/f,Gfap-cre/+^, *n* = 7). Data are mean ± SEM. Student’s *t*-test in the dishabituation task and two-way ANOVA with Holm-Sidak *post hoc* comparison between trial #1 and #4 in the habituation task. **p < 0.01, ***p < 0.001.

Since odor discrimination inability has been reported in other genetically modified mice with reduced number of newly generated GCs (Gheusi et al., 2000; Bath et al., 2008; Zou et al., 2012), reduced GC survival during the early postnatal OB development may explain the impaired odor discrimination in CPEB4-KO mice. Because CPEB4 is expressed in all bulbar neurons (Tseng et al., 2017) and because of lack of a Cre mouse line with specific deletion of *Cpeb4* in neonate-born GCs, we used two cKO mouse genotypes for the discrimination task: *Gad65-Cre* to ablate *Cpeb4* in all GABAergic interneurons (CPEB4-cKO^GAD^) and *Gfap-Cre* to remove *Cpeb4* in SVZ-derived interneurons (CPEB4-cKO^GFAP^; Garcia et al., 2004). Although GFAP is also expressed in radial glia and astrocytes (Bailey et al., 1999), the *Gfap-Cre* transgenic mouse (line 73.12) used here has been used previously to manipulate gene expression for studying SVZ adult neurogenesis (Garcia et al., 2004; Cushman et al., 2012). By crossing with a reporter mouse, green fluorescent protein (GFP)-labeled SVZ-derived GCs appear sparsely in the OB around P14 and continuously increase until reaching a plateau (Cushman et al., 2012). In CPEB4-cKO^GFAP^ mice, we also found that CPEB4-deficient GCs are distributed in the deeper GCL (Tseng et al., 2017) where is also the site BrdU-labeled SVZ-derived GCs accumulated (Lemasson et al., 2005). In CPEB4-cKO^GAD^ mice, we found very few CPEB4-positive cells left in the GCL (Tseng et al., 2017). Moreover, only CPEB4-cKO^GAD^ mice recapitulate the olfactory defects, including increased apoptotic GCs and OB size during early postnatal period (i.e., 2 weeks after birth; Tseng et al., 2017). Consistent with the findings in global CPEB4-KO mice, CPEB4-cWT and -cKO^GAD^ mice exhibited normal habituation (cWT, *F*_(3,27)_ = 11.376, *p* < 0.001; cKO^GAD^, *F*_(3,27)_ = 9.108, *p* < 0.001). As expected, CPEB4-cWT mice could discriminate the novel odor (*p* < 0.001) but their cKO^GAD^ littermates failed (*p* = 0.24; Figure 2A). By contrast, CPEB4-cKO^GFAP^ mice, with normal adult neurogenesis and OB size (Tseng et al., 2017), performed similar with their cWT littermates in both habituation (cWT, *F*_(3,18)_ = 5.213, *p =* 0.009; cKO^GFAP^, *F*_(3,18)_ = 7.562, *p* = 0.002) and dishabituation tasks (cWT, *p* = 0.003; cKO^GFAP^, *p* = 0.005; Figure 2B).

**Figure 3:**
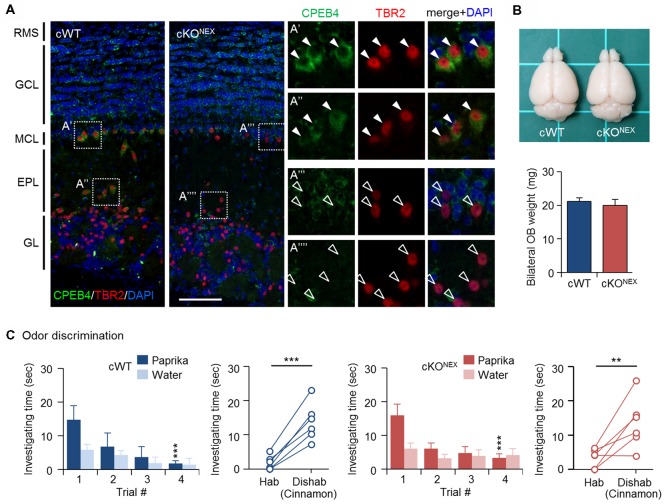
CPEB4-cKO^NEX^ mice with loss of CPEB4 in projection neurons show normal olfactory discrimination. **(A)** Immunostaining of CPEB4 and T-box brain protein 2 (TBR2) in coronal olfactory bulb (OB) sections prepared from CPEB4-cWT (*Cpeb4*^f/f,+/+^) and -cKO^NEX^ (*Cpeb4*^f/f,*Nex-cre*/+^) adult mice. The magnified images of selected areas show mitral cells (MCs; **A′,A″′**) and tufted cells (TCs; **A″,A″″**). RMS, rostral migratory stream; GCL, granule cell layer; MCL, mitral cell layer; EPL, external plexiform layer; GL, glomerular layer. Scales, 100 μm. **(B)** Dorsal view of adult cWT and cKO^NEX^ brains. Mean OB weight in cWT and cKO^NEX^ female mice (*n* = 5 for both groups). **(C)** Performance of cWT and cKO^NEX^ female mice (*n* = 6 per group) in the odor discrimination test. Data are mean ± SEM. Student’s *t*-test in the dishabituation task and two-way ANOVA with Holm-Sidak *post hoc* comparison between trial #1 and #4 in the habituation task. **p < 0.01, ***p < 0.001.

To exclude the possible contribution from CPEB4 in projection neurons, MCs and TCs, we used the *Nex-Cre* mouse line to deplete CPEB4 expression in excitatory neurons (Goebbels et al., 2006). Immunostaining results confirmed the absence of CPEB4 in TBR2-positive MCs in the MC layer (Figures 3A′,A″′) and TCs in the external plexiform layer (EPL) (Figures 3A″,A″″) of CPEB4-cKO^NEX^ OBs (Figure 3A). The OB size and weight (Figure 3B) in CPEB4-cKO^NEX^ mice were normal in comparison to their cWT littermates (*p* = 0.61, Figure 3B), suggesting projection neuron-specific CPEB4 deficiency did not affect OB growth. Accordingly, both CPEB4-cWT and-cKO^NEX^ mice exhibited normal habituation (cWT, *F*_(3,15)_ = 7.671, *p =* 0.002; cKO^NEX^, *F*_(3,15)_ = 6.752, *p* = 0.004) and discrimination (cWT, *p* = 0.001; cKO^NEX^, *p* = 0.009). Taken together, CPEB4 in projection neurons and SVZ-derived interneurons is dispensable for olfactory discrimination.

### SVZ-Derived GCs Eventually Rescue OB Size but Not Discrimination Ability in 6-Month-Old KO Mice

**Figure 4:**
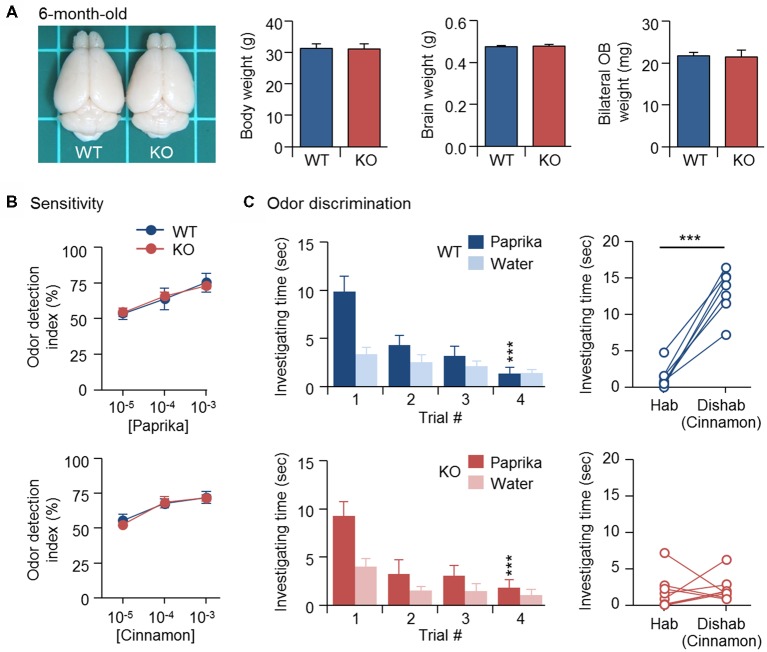
Recovery of OB size could not rescue olfactory dysfunction in 6-month-old CPEB4-KO mice. **(A)** Dorsal view of CPEB4-WT and -KO brains. Mean body, brain and OB weight of 6-month-old WT and KO female mice (*n* = 9 for both groups). CPEB4-WT (*n* = 7) and -KO (*n* = 8) female mice at age 6 months were tested for **(B)** olfactory sensitivity and **(C)** odor discrimination. Data are mean ± SEM. Student’s *t*-test in the dishabituation task and two-way ANOVA with Holm-Sidak *post hoc* comparison between trial #1 and #4 in the habituation task. ***p < 0.001.

Newborn GCs derived from adult SVZ continue to replace existing GCs and maintain the cellular component of bulbar circuits (Imayoshi et al., 2008). Similar to CPEB4 deficiency, naris closure-deprived olfactory input during the early postnatal period (from P1 to P20) also reduced OB size and neonate-born GC number, which could be rescued by SVZ-dependent adult neurogenesis after naris reopening (Cummings et al., 1997). Thus, we expected that normal adult neurogenesis in CPEB4-KO mice, given sufficient time, would eventually restore their OB size. Indeed, body weight (*p* = 0.95), brain size (*p* = 0.76) and OB weight (*p* = 0.86) were comparable between CPEB4-KO mice at age 6 months and their WT littermates (Figure 4A), and their olfactory sensitivity was normal (Figure 4B). Although 6-month-old CPEB4-KO mice remained normal habituation (WT, *F*_(3,18)_ = 9.579, *p* < 0.001; KO, *F*_(3,21)_ = 41.977, *p* < 0.001), the restoration of OB size by adult neurogenesis did not rescue their ability to recognize a novel odor in the dishabituation trial (*p* = 0.71; Figure 4C). Hence, our results suggest that GCs born in the early postnatal OB are essential to construct the local inhibitory circuits to support life-long odor discrimination.

## Discussion

In this study, we used mice with global or GABAergic ablation of *Cpeb4* gene to affect the survival of only neonate-born GCs (Tseng et al., 2017) and found that mice had an inability to discriminate odors. Because the continuous replenishment of GCs from SVZ neurogenesis eventually restored the bulbar size but not olfactory function in 6-month-old KO mice, we suggest the inhibitory circuits constructed by CPEB4-dependent neonate-born GCs plays an indispensable role in olfactory discrimination.

CPEB4 is widely expressed in the brain, including the cortex, hippocampus and amygdala, besides the OB (Tsai et al., 2013). Unlike its close family members CPEB2 and CPEB3, which play regulatory roles in hippocampus-dependent long-term memory and synaptic plasticity (Chao et al., 2013; Lu et al., 2017), CPEB4 seems to be dispensable for hippocampus-dependent plasticity and memory. CPEB4-KO mice perform normally in the battery of behavior tasks including open-field, elevated plus maze, rotarod, contextual fear conditioning and Morris water maze (Tsai et al., 2013). So far, OB hypoplasia is the only neuronal defect found in CPEB4-KO mice (Tseng et al., 2017). Because of normal odor discrimination in CPEB4-cKO^NEX^ mice (Figure 3C), the role of CPEB4 in either projection neurons or excitatory afferent fibers from the piriform cortex to GCs is not required for olfactory discrimination. By contrast, CPEB4-cKO^GAD^ mice showed impaired odor discrimination (Figure 2A), so the loss of CPEB4 in GABAergic neurons is responsible for such a defect. Notably, CPEB4 expression is not restricted to bulbar GCs, so it may function in GABAergic neurons outside of OBs to indirectly influence the excitability of GCs. It is known that GABAergic afferents originating from the basal forebrain innervate the bulbar GCL (Gaykema et al., 1990; Gracia-Llanes et al., 2010) and evoke inhibitory responses in GCs (Nunez-Parra et al., 2013). Moreover, chemogenetic disruption of this centrifugal inhibitory input increased GC excitability and thereby diminished the ability of mice to discriminate structurally similar odors [i.e., esters differing by one carbon: ethyl heptanoate (C7) vs. ethyl octanoate (C8)] but not more dissimilar odors (i.e., esters differing by two carbons: ethyl hexanoate [C6] vs. ethyl octanoate [C8]; Nunez-Parra et al., 2013). Both CPEB4-KO and CPEB4-cKO^GAD^ mice failed to distinguish complex odor mixtures (Figures 1C, 2A), so aberrant inhibitory signaling from the basal forebrain, if any in the absence of CPEB4, is unlikely responsible for their discrimination dysfunction. Furthermore, depletion of CPEB4 in SVZ-derived GCs did not affect adult neurogenesis (Tseng et al., 2017) and olfactory discrimination in CPEB4-cKO^GFAP^ mice (Figure 2B). Together, we suggest that the reduction of neonate-born GCs accounts for discrimination dysfunction in CPEB4-KO mice. However, in the absence of direct evidence from mice with specific elimination of CPEB4 in neonate-born GCs, we cannot exclude the possibility that CPEB4 may function in other unidentified centrifugal inhibitory inputs to influence odor discrimination.

Olfactory discrimination depends on, in part, the GC inhibitory input to MCs/TCs for refining neuronal representation of odors. Therefore, manipulating the activity of GCs (Abraham et al., 2010) or adult-born GCs (Alonso et al., 2012) in mice affects discrimination sensitivity in reward-forced odor discrimination tasks. Moreover, discrimination learning (Alonso et al., 2006) and perceptual learning (Moreno et al., 2009) enhance the survival of adult-born GCs. Blocking neurogenesis by infusion of mitotic inhibitor cytosine arabinoside (AraC) in the SVZ before or during perceptual learning (i.e., odorant exposure) prevents the increase in inhibitory activity, thereby abolishing learning-acquired ability to distinguish chemically similar odorants (Moreno et al., 2009). While adult-born GCs improve learning-associated discrimination ability, spontaneous odor discrimination remains unaffected by ablation of neural stem cells in the adult SVZ by focal irradiation (Lazarini et al., 2009; Díaz et al., 2017) or AraC infusion (Breton-Provencher et al., 2009). Although the definite role of newborn GCs in fine-tuning which aspect of olfactory function remains active investigation, the results from CPEB4-KO mice indicate the indispensable role of neonate-born GCs in spontaneous odor discrimination.

Several genetically engineered mice with defective adult neurogenesis also show OB hypoplasia and impaired spontaneous odor discrimination (Gheusi et al., 2000; Bath et al., 2008; Zou et al., 2012). However, such a dysfunction may be accumulated from early postnatal OB development, when proliferation and apoptosis are already in operation to select locally neonate-born GCs (Fiske and Brunjes, 2001; Tseng et al., 2017) or early-born SVZ-derived GCs (De Marchis et al., 2007; Kelsch et al., 2007). Because these mice exhibited defects in adult neurogenesis, their early postnatal neurogenesis has not been examined. In contrast, CPEB4-KO mice with normal adult neurogenesis led us to uncover that insufficient BDNF signaling also affects the survival of neonate-born GCs (Tseng et al., 2017) besides adult-born GCs (Bath et al., 2008). Thus, impaired spontaneous odor discrimination in *Bdnf*^+/–^, *Trkb*^+/–^ and *Bdnf*^Val66Met^-knockin mice (Bath et al., 2008) may result from increased GC apoptosis in early postnatal neurogenesis.

A transient naris occlusion (from P5 to P19) in mice moderately impairs odor discrimination ability in adulthood only by mice housed in an odor-deprived environment after naris reopening; mice housed in an odor-enriched environment do not show impaired ability (Kato et al., 2012). Although the loss of olfactory afferents reduces the survival of interneurons (Fiske and Brunjes, 2001; Tseng et al., 2017), P5–P19 naris occlusion probably only moderately decreases the number of neonate-born GCs because the most active proliferation (Lemasson et al., 2005) and elimination of neonate-born GCs occur very early (i.e., P0 to P3) after birth (Tseng et al., 2017). Hence, the remaining neonate-born GCs after a P5–P19 sensory deprivation may be sufficient to construct odor discrimination circuits whose connections could be further strengthened under an odor-enriched environment after naris reopening.

Because neonate- and adult-born GCs are differentially distributed in the GCL (Lemasson et al., 2005; Kelsch et al., 2007), they likely construct different inhibitory circuits to modulate distinct olfactory function. Accordingly, newly generated adult-born GCs and preexisting mature GCs show different responses to novel and familiar odors (Magavi et al., 2005). Adult neurogenesis-supplied GCs eventually restored OB size but not discrimination ability in 6-month-old CPEB4-KO mice (Figures 4A,C). Hence, odor discrimination impairment in CPEB4-KO mice may result from the reduction of the reduction of neonate-born GC-wired local circuits rather than a decrease in total GC number. The bulbar wiring circuit is composed of heterogeneous GC subtypes, which are identified according to their morphological, electrophysiological and/or molecular differences as well as topological distribution in the GCL (Takahashi et al., 2018). Deletion or inhibition of single subtype GCs (i.e., trophoblastic glycoprotein 5T4-positive or calretinin-positive GCs) causes significant olfactory dysfunctions (Takahashi et al., 2016; Hardy et al., 2018), suggesting their functions in the olfactory circuit cannot be substituted by other GC subtypes. Further investigations are needed to understand the spatial and temporal interaction among heterogeneous populations of OB interneurons for processing odor discrimination.

## Author Contributions

C-ST designed and conducted the experiments, analyzed data and wrote the manuscript. S-JC provided the Cre line to generate cKO^NEX^. Y-SH designed and supervised the study, co-wrote the manuscript and is responsible for its content.

## Conflict of Interest Statement

The authors declare that the research was conducted in the absence of any commercial or financial relationships that could be construed as a potential conflict of interest.

## Supplementary Material

FIGURE S1 Impaired odor discrimination in CPEB4-KO male mice. CPEB4-WT and -KO male littermates (~3-month-old) were used for assays (*n* = 6 per group). Habituation (Hab, bar graphs) test of the mean time mice spent sniffing paprika vs. water for four consecutive trials to become familiar with the odor and gradually reduce investigation interest. Discrimination test: in the 5th dishabituation (Dishab) trial, mice were exposed to a novel cinnamon odor. The time mice spent investigating the two odors was recorded. Data are mean ± SEM. Student’s *t*-test in dishabituation tasks and two-way ANOVA with Holm-Sidak *post hoc* comparison between trial #1 and #4 in habituation tasks, ****p* < 0.001.
