# Supplementary figures and images for: CPEB4-Dependent Neonate-Born Granule Cells Are Required for Olfactory Discrimination
Source: Front Behav Neurosci. 2019 Jan 23;13:5. doi: 10.3389/fnbeh.2019.00005 (PMC6351472; doi:10.3389/fnbeh.2019.00005)

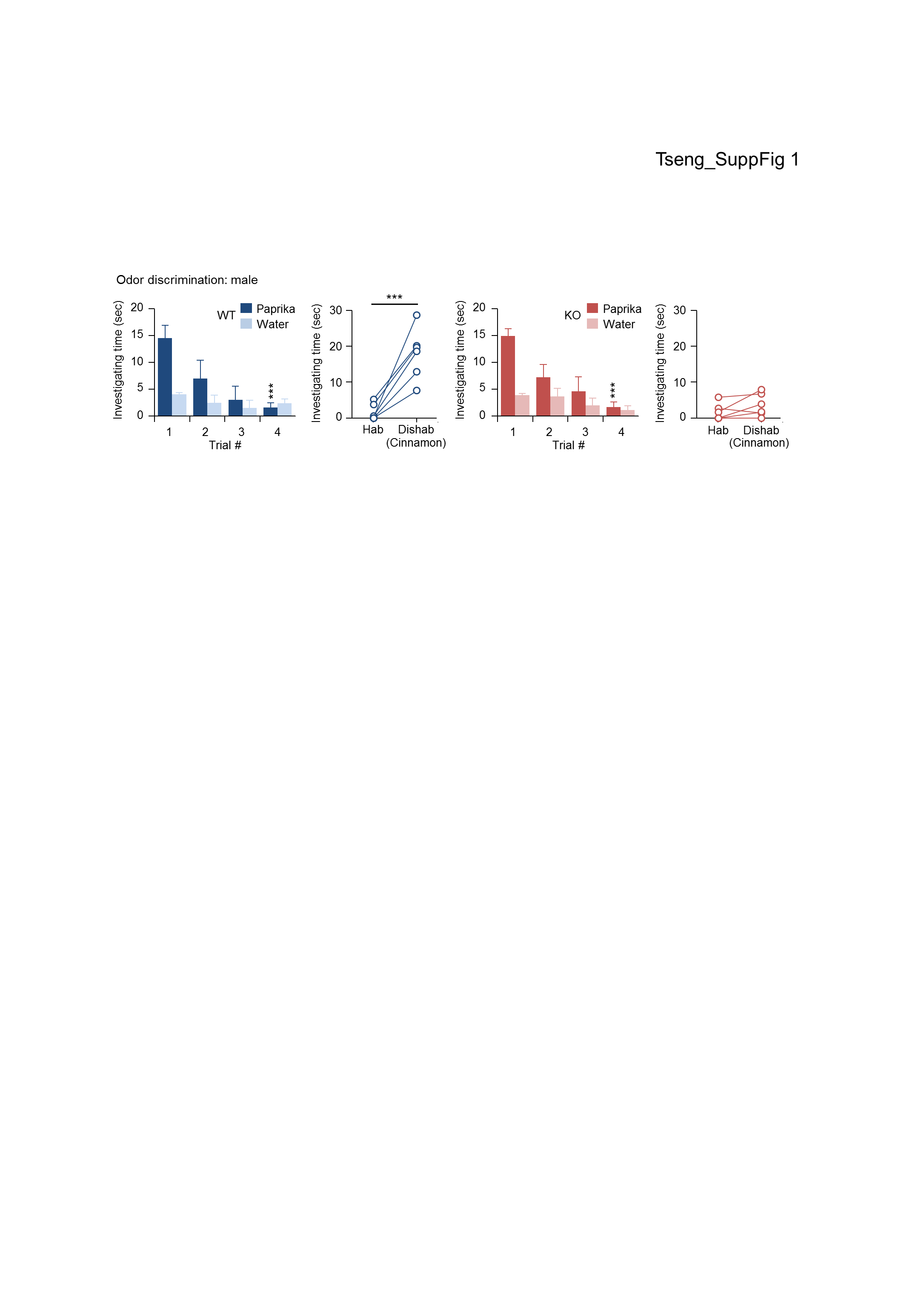

Supplement: FIGURE S1 — Impaired odor discrimination in CPEB4-KO male mice. CPEB4-WT and -KO male littermates (~3-month-old) were used for assays (n = 6 per group). Habituation (Hab, bar graphs) test of the mean time mice spent sniffing paprika vs. water for four consecutive trials to become familiar with the odor and gradually reduce investigation interest. Discrimination test: in the 5th dishabituation (Dishab) trial, mice were exposed to a novel cinnamon odor. The time mice spent investigating the two odors was recorded. Data are mean ± SEM. Student’s t-test in dishabituation tasks and two-way ANOVA with Holm-Sidak post hoc comparison between trial #1 and #4 in habituation tasks, ***p < 0.001. [file Image_1.tif]
